# The Neoantigen Landscape of Mycosis Fungoides

**DOI:** 10.3389/fimmu.2020.561234

**Published:** 2020-11-23

**Authors:** Arunima Sivanand, Dylan Hennessey, Aishwarya Iyer, Sandra O’Keefe, Philip Surmanowicz, Gauravi Vaid, Zixuan Xiao, Robert Gniadecki

**Affiliations:** Division of Dermatology, Department of Medicine, University of Alberta, Edmonton, AB, Canada

**Keywords:** neoantigen, cutaneous T-cell lymphoma, mycosis fungoides, immunogenicity, immunotherapy

## Abstract

**Background:**

Mycosis fungoides (MF) is the most common cutaneous T-cell lymphoma, for which there is no cure. Immune checkpoint inhibitors have been tried in MF but the results have been inconsistent. To gain insight into the immunogenicity of MF we characterized the neoantigen landscape of this lymphoma, focusing on the known predictors of responses to immunotherapy: the quantity, HLA-binding strength and subclonality of neoantigens.

**Methods:**

Whole exome and whole transcriptome sequences were obtained from 24 MF samples (16 plaques, 8 tumors) from 13 patients. Bioinformatic pipelines (Mutect2, OptiType, MuPeXi) were used for mutation calling, HLA typing, and neoantigen prediction. PhyloWGS was used to subdivide malignant cells into stem and clades, to which neoantigens were matched to determine their clonality.

**Results:**

MF has a high mutational load (median 3,217 non synonymous mutations), resulting in a significant number of total neoantigens (median 1,309 per sample) and high-affinity neoantigens (median 328). In stage I disease most neoantigens were clonal but with stage progression, 75% of lesions had >50% subclonal antigens and 53% lesions had CSiN scores <1. There was very little overlap in neoantigens across patients or between different lesions on the same patient, indicating a high degree of heterogeneity.

**Conclusions:**

The neoantigen landscape of MF is characterized by high neoantigen load and significant subclonality which could indicate potential challenges for immunotherapy in patients with advanced-stage disease.

## Introduction

Mycosis fungoides (MF) is the most common type of cutaneous T-cell lymphoma (CTCL) that develops from clonotypically diverse malignant T-cell precursors seeding the skin (1, 2). Prognosis in the early stages (T1-T2, patches and plaques) is excellent, however the development of tumors (T3) or erythroderma (T4) is associated with a significant decrease in survival (3, 4). Despite intensive research, MF remains incurable and treatments for advanced disease are mostly palliative (4).

There is robust evidence that MF is an immunogenic tumor and that the immune system is an essential factor limiting its progression [reviewed in ref. (5)]. It has been well documented that iatrogenic immunosuppression causes a catastrophic dissemination of MF (6, 7). Many current therapies (interferons, imiquimod, extracorporeal photopheresis and allogeneic stem cell transplant) are considered to act primarily *via* stimulation of the antitumor immunity (8–11). However, the experience with immune checkpoint inhibitors has been disappointing in MF (5). The literature comprising approximately 50 cases of MF treated with various immune checkpoint inhibitors reports response rates ranging from 9% to 56% with only a few documented complete remissions (12–16). Of the few anticancer vaccine studies in CTCL, response rates have ranged from 33% to 50% (17–19). Those rather discouraging results are surprising in view of the fact that MF is a mutationally rich tumor with a mutation load in the range of 500–4,500 somatic mutations/genome (20). The number of mutations is usually correlated with the number of neoantigens and consequently the immunogenicity of the cancer, which is predictive for immune checkpoint inhibitor efficacy (21–23).

It has recently been suggested that in addition to mutational load and the number of neoantigens, tumor heterogeneity has a major impact on the ability of the host immune system to mount an effective antitumor defense. Neoantigens can be classified as clonal (present on all cancer cells) or subclonal (present only on a subset (subclones) of cancer cells) (21). A high clonal neoantigen burden, for instance in malignant melanoma, favors effective immune surveillance, response to immunotherapy and significantly prolonged survival (21). In contrast, a tumor with a branched subclonal structure will be poorly recognized by the immune system, even if the mutation load is high, as documented for some immunotherapy-resistant tumors such as glioblastomas (24).

To better understand the potential for immunotherapeutic approaches in MF we studied the landscape of neoantigen expression in this malignancy. Using whole transcriptome and whole exome sequencing, we determined the pattern of neoantigens in early lesions of patches and plaques and compared them to those of clinically advanced disease. We show that disease progression is correlated with an increase in mutational load and the number of neoantigens. However, advanced lesions of MF exhibit a high proportion of subclonal neoantigens which may limit the efficacy of immunotherapies.

## Materials and Methods

### Materials, Sequencing, Datasets

**Figure 1:**
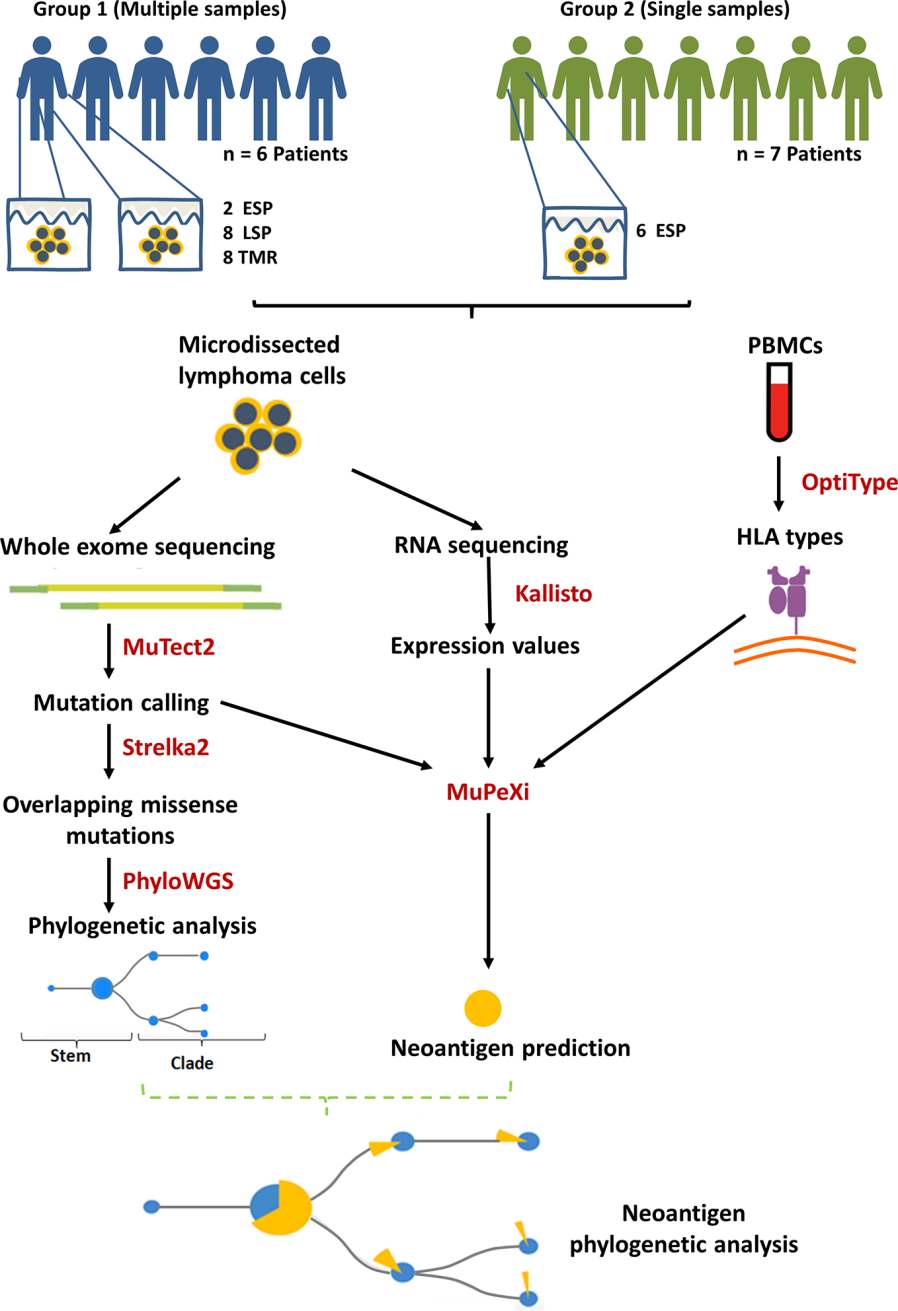
Summary of methods and study design. Biopsies of lesional skin and blood were obtained. 13 mycosis fungoides (MF) patients were divided into group 1 (multiple samples) and 2 (single samples) according to the number of biopsies contributed. The lesions were categorized according to the clinical stage and the morphology of the lesion: ESP (early stage plaques, i.e. MF plaques in stage I), LSP and TMR (respectively, late stage plaques and tumors biopsied from patients in stage ≥IIB). MuPeXi was used to predict neoantigens. For clonality analysis we used mutation data obtained from MuTect2 and Strelka2, as described previously (20). Predicted neoantigens were mapped to the clades and stems of the phylogenetic trees constructed using PhyloWGS (20).

Institutional ethics approval was obtained under the application HREBA.CC-16-0820-REN1. We performed whole exome sequencing (WES) and whole transcriptome sequencing (WTS) of 24 MF samples (16 plaque, 8 tumor) and matched peripheral blood mononuclear cell (PBMC) in 13 patients (patient characteristics in **Supplementary Table S1**). DNA and RNA sequencing libraries were prepared from tumor cell clusters microdissected from skin biopsies using laser capture microdissection and sequenced as described previously (20, 25) (**Figure 1**). The mean sequencing depth across samples was 162.62x (individual sequencing depths in **Supplementary Table S2**). Additional datasets comprised sequencing data (study characteristics in **Supplementary Table S3**) published by McGirt et al. (5 whole genome sequences (WGS) from 5 patients with MF) (26) and by Choi et al. (31 WES from 31 patients with Sézary syndrome) (27).

### Identification of Neoantigens

Bioinformatics analysis involved a series of pipelines shown in **Figure 1**. GATK (v4.0.10) best practices guidelines (28) were used to process the initial WES fastq files. Reads were aligned to the hg38 reference genome. MuTect2 (v2.1) was used for variant calling to identify missense and indel mutations. OptiType (v1.3.1) (29) was used with default settings to predict class I human leukocyte antigen (HLA) types from WES of PBMC for the corresponding samples. Kallisto (v0.45.0) (30) was used to process the raw RNA fastq files with the bootstrapping function set to 500 to obtain the variance and expression level. The outputs of these pipelines (.vcf files from MuTect2, HLA types from Optitype and.tsv files from Kallisto) were imported into MuPeXi (v1.2) (31) to predict neoantigenic peptides (8-11 amino acids long). Of note, MuPeXi penalizes neopeptides that are identical to their wildtype, as these are likely not immunogenic due to central tolerance. Neopeptides are prioritized based on their dissimilarity to their unmutated form. NetMHCpan 4.0 (32) (incorporated in MuPeXi pipeline) was used to predict peptide binding affinities to up to 6 patient-specific HLA types.

### Neoantigen Filtering

We will refer to the raw output of prediction software as ‘putative neoantigens’ and the result once filtering criteria is applied as ‘filtered neoantigens’. Our filtering criteria included: *(1)* Mutant peptide binding strength, defined as eluted ligand (EL) likelihood percentile rank ≦0.5%, *(2)* RNA expression level >0.1 transcripts per million (TPM) (33), *(3)* Top peptide, applied last to group all predictions arising from the same mutation (chromosome and genomic position) and select the peptide with the lowest binding strength. While all peptides <0.5% rank are generally considered to be strong binders (34), we further divided these into high strength binders (<0.05%rank), intermediate strength binders (0.05≧%rank<0.15) and low strength binders (0.15≧%rank≦0.5).

### Mutant Peptide Characterization

To further characterize mutant peptides, we identified the most frequently overlapping peptides between samples. We then used the mutant peptide sequence to search the IEDB database (35) for homologous peptides that were known immune epitopes. We searched for exact matches and if none were found, we reduced the threshold to blast >90%. If a known epitope was found, we further searched the Uniprot database (36) for details of the gene encoding the protein, and the protein function.

### Neoantigen Clonality Analysis

For phylogenetic analysis, Strelka2 (v2.9.10) (37) was used for mutation calling to identify missense mutations that overlapped with those called by MuTect2. TitanCNA (38) was used to predict copy number aberrations (CNA). Default parameters were used except for alphaK which was changed to 2,500 as recommended for WES data. PhyloWGS (v1.0-rc2) (39) was used to build phylogenetic trees by clustering missense mutations using CNA. The stem and clade mutations producing neoantigens were then highlighted on the phylogenetic trees to determine the clonality of the neoantigens.

### Cauchy-Schwarz Index of Neoantigens (CSiN)

CSiN combines neoantigen load, neoantigen clonality and immunogenicity in a single score and is believed to reflect the sensitivity of the tumor to immunotherapy (40). We used transcripts per million (TPM) counts calculated by Kallisto (30) for RNA expression, and neoantigen binding strengths calculated by MuPeXI (31). We used seven binding strength thresholds (%rank) of 0.375, 0.5, 0.625, 0.75, 1.25, 1.75, and 2. Only neoantigens that had an expression ≥ 1 TPM and were also produced by the top 500 most common variants (variants with the highest variant allele frequency) were included in the CSiN score calculation.

### Data Visualization

Visual data representations were created using the R package beeswarm (www.cbs.dtu.dk/~eklund/beeswarm/), GraphPadPrism (v8.3.0, www.graphpad.com), jvenn (41), Venn Diagram Tool (http://bioinformatics.psb.ugent.be/webtools/Venn/), PhyloWGS (39) and Microsoft Excel.

## Results

### Tumor Mutation Burden in MF Is Dominated by Frameshift Mutations

**Figure 2:**
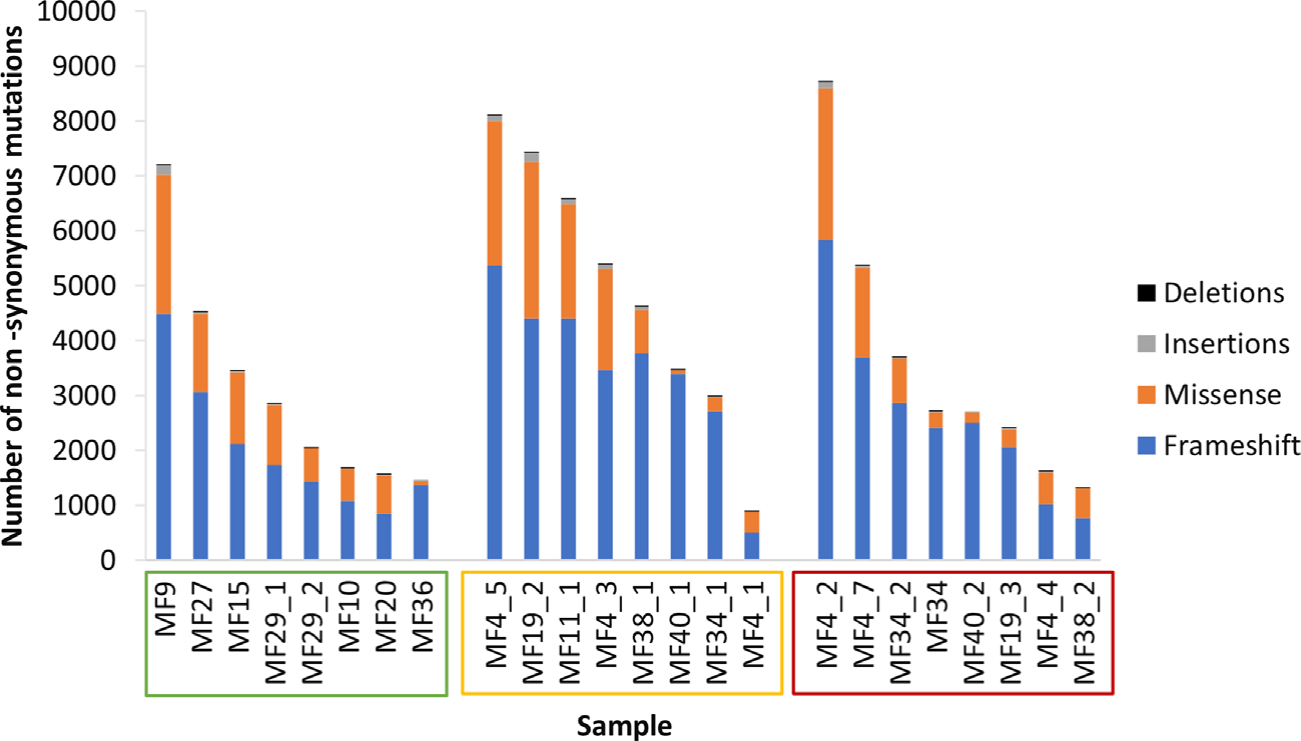
Tumor mutation burden. Samples are arranged in descending order of tumor mutation burden (TMB) and sample names enclosed in boxes with colors corresponding to the lesion type - early stage plaque (green), late stage plaque (yellow) and tumor (red).Frameshift mutations comprise the majority of non-synonymous mutations.

Early-stage MF (IA-IIA) is characterized by thin cutaneous lesions of patches and plaques (T1-T2). The emergence of tumors (T3) heralds progression to the advanced stage IIB. It is important to note that most advanced-stage patients may exhibit plaques persisting from the early stages in addition to the stage-defining tumors. To capture the impact of disease stage on mutation burden and neoantigen expression we classified biopsies into the following categories: early stage plaques (ESP), i.e. the lesions T1 and T2 (patches or plaques) obtained from patients in stage IA-IB, and late stage plaques (LSP) and matched tumors (TMR) from patients in a clinical stage ≥ IIB **(** **Figure 1** **)**. In those lesions, we determined tumor mutation burden (TMB) defined as the number of non-synonymous mutations producing neoantigens. The median TMB was 3,217 mutations per sample, or 35 mutations/kB consisting primarily of frameshift mutations (70.3%), in-frame missense mutations (28.4%), insertions (1.1%) and deletions (0.2%) **(** **Figure 2** **)**. The median TMB in ESP was 2,455 (range 1,440-7,198), and its upper range increased in LSP (median 5014, range 890-8,697) and in TMR (median 2,697; range 1306-8,722).

### Increase in Neoantigen Load During Disease Progression

**Figure 3:**
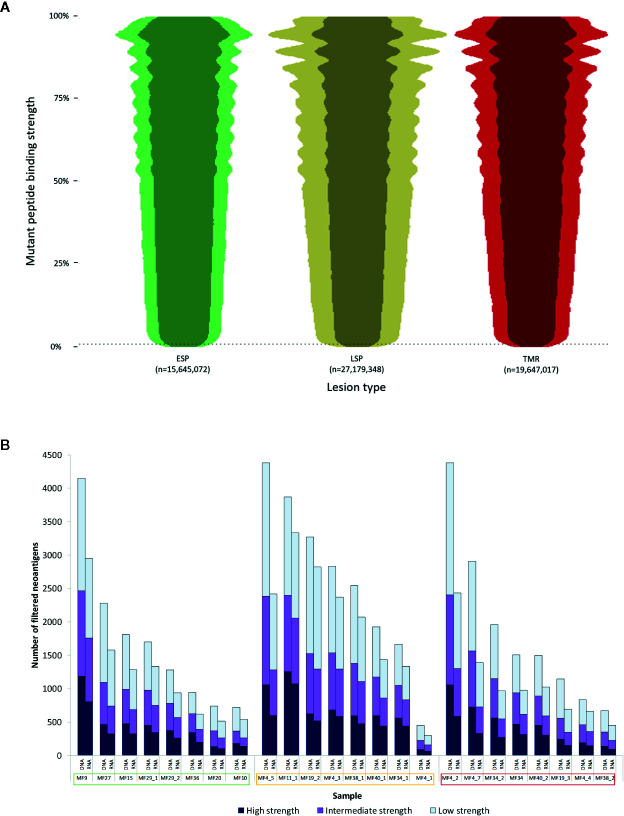
Characterization of neoantigens in mycosis fungoides (MF). **(A)** Beeswarm plot representation of putative neoantigens prior to filtering. Due to the extensive size of the dataset, a random 1% of all data points were plotted to demonstrate the overall distribution and density of the data. The vertical axis shows mutant peptide binding strength as a percentile rank, with lower values representing increasingly strong binding peptides to human leukocyte antigen (HLA) types. 0.5% rank (dashed line) represents the commonly used cutoff below which peptides are considered strong enough binders to be neoantigens. The width of each plot is proportional to the number of neoantigens at each binding strength. Overall, early stage plaques (ESP) lesions had fewer neoantigens compared to late stage plaques (LSP) and matched tumors (TMR). The darker shade within each plot represents the neoantigens expressed in RNA (TPM>0.1). **(B)** Neoantigen load before and after applying the RNA filter. For each sample, the “DNA” column has all filters applied with the exception of the RNA filter. The median number of filtered neoantigens per sample was 1,309. The “RNA” column has all filters including the RNA filter (expression >0.1 TPM) applied. On average 70% of predictions were expressed in RNA. Samples names enclosed in boxes with colors corresponding to the lesion type - early stage plaque (green), late stage plaque (yellow) and tumor (red).

When examined by lesion type, patients with advanced disease had a greater number of putative neoantigens compared to early stage plaques (LSP - 27,179,348, TMR - 19,647,017 vs ESP - 15,645,072), though this was not statistically significant (P=0.368) (**Figure 3A**). There was no difference in median binding strength between ESP (median 56%), LSP (median 57%) and TMR (median 58%).

Filtering putative neoantigens is necessary to narrow down epitopes that are most likely expressed in patients. When we applied all filters (“RNA” column in **Figure 3B**), an average of 70% of predicted neoantigens were expressed at the RNA level (median neoantigens per sample was 1,309). A median of 328 were high strength binders (<0.05%rank), 376 were intermediate strength binders (0.05≧%rank<0.15) and 540 were low strength binders (0.15≧%rank≦0.5).

**Figure 4:**
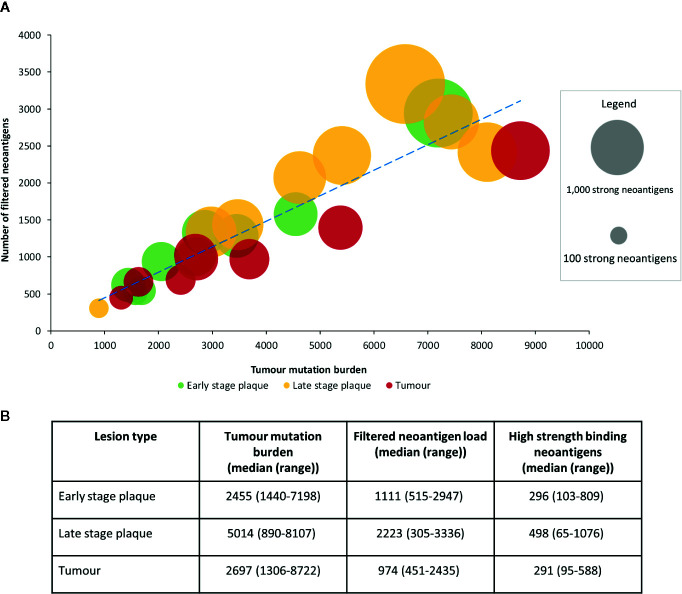
Relationship between tumor mutation burden and filtered neoantigen load in mycosis fungoides (MF). **(A)** A strong positive linear association (r=0.92, blue dashed trendline) was observed between tumor mutation burden and filtered neoantigen load. Each bubble represents a single sample, with its size proportional to the number of high strength neoantigens (<0.05%rank). A positive linear association was also observed between tumor mutation burden and the high strength neoantigen load (r=0.81). **(B)** Mutations and neoantigen numbers by lesion type.

We further compared the association between tumor mutation burden and the filtered neoantigen load (**Figure 4A**), which showed a strong positive linear relationship (r=0.92). The tumor mutation burden also demonstrated a positive linear relationship with the number of high strength neoantigens (r=0.81). The tumor mutation burden, filtered neoantigen load and number of high strength neoantigens are summarized in **Figure 4B**.

Comparing our data to the two previous CTCL studies of McGirt et al. (26) and Choi et al. (27), we found that our dataset had a much higher neoantigen count (total 54,073,746 vs 615,761 in Choi et al. (27) or 135,042 in McGirt et al. (26), **Supplementary Figure S1** **)**. Tumor mutation burden and neoantigen count is influenced by various factors including CTCL subtype, methodology and sequencing depth. Choi’s samples, as they were all Sezary Syndrome, permitted the use of cell sorting which improves tumor cell fraction (the percent of sample composed of tumor cells). While ours and McGirt’s study comprised mycosis fungoides samples, our use of laser capture microdissection (instead of whole biopsies) increased tumor cell fraction. Additionally, the use of whole exome sequencing with greater sequencing depth in ours and Choi’s studies increased sensitivity to mutations compared to McGirt’s whole genome sequencing (which includes non-coding intronic regions) at lower sequencing depth. Details of the 3 studies are included in **Supplementary Tables S2** and **S3**.

### Increase in Proportion of Subclonal Neoantigens in Advanced MF

**Figure 5:**
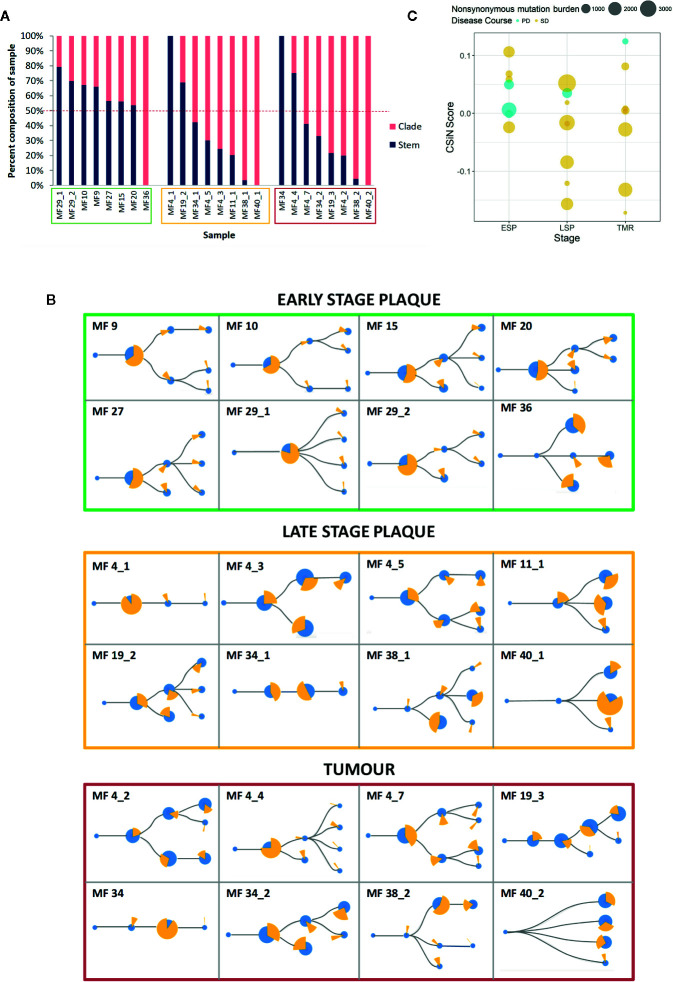
Clonality of neoantigens in mycosis fungoides (MF). **(A)** Proportion of stem and clade missense mutations producing putative neoantigens. Sample codes are enclosed in boxes with colors corresponding to the lesion type - early stage plaque (green), late stage plaque (yellow) and tumor (red). The dashed red line represents the 50% mark that distinguishes whether the majority of the sample is composed of stem or clade neoantigens. Early stage plaques have a greater proportion of stem mutations producing neoantigens compared to late stage plaques and tumors where more clade mutations produce neoantigens. **(B)** Phylogenetic trees with putative neoantigen analysis. The size of the blue circles represents the proportion of missense mutations that comprise each node. ‘Stem’ nodes are those present prior to branching which then produces ‘clade’ nodes. The yellow pie chart in whole represents all neoantigens from the sample. Each slice of the pie chart represents the proportion of neoantigens originating from a node. With advancing disease stage, a greater proportion of neoantigens originate from clade mutations. **(C)** Cauchy-Schwarz index of Neoantigens (CSiN) scores of MF samples. The bubble plot shows individual CSiN scores and the number of non-synonymous mutations in ESP, LSP and TMR samples. Samples from patients who progressed in disease stage are colored in blue. Median CSiN scores are: ESP (0.027822, n=8), LSP (−0.01709, n=8) and TMR (0.004234, n=8).

To determine the subclonality of the neoantigens we first constructed phylogenetic trees showing the subclonal architecture of MF, as described previously (20). Then we mapped the neoantigens to the stem and clades, the latter representing the subclonal neoantigens (**Figure 5A**). This analysis demonstrated an increasing branching with a higher proportion of clade neoantigens in advanced lesions, as demonstrated in LSP (median 62% clade neoantigens) and TMR (median 70%), compared to ESP (median 39%) (**Figures 5A, B**).

The Cauchy-Schwarz index of Neoantigens (CSiN) reduces the number of neoantigens, their clonality and immunogenicity in the sample to a single number (40). CSiN has been argued to out-perform existing metrics as a biomarker of tumor immunogenicity and response to immune checkpoint inhibitors across different neoplasms (40). The CSiN scores of our samples are shown in **Figure 5C**. As expected, there was no significant correlation between CSiN and non-synonymous mutational burden (P = 0.637), however a greater proportion of early lesions (ESP) had the higher, advantageous CSiN scores >1 compared to the late-stage lesions (LSP and TMR). However, higher CSiN scores did not predict more favorable prognosis (defined as lack of stage progression) in our cohort (**Figure 5C**, regression analysis, P = 0.142).

### Neoantigen Overlap and Peptide Identity

**Figure 6:**
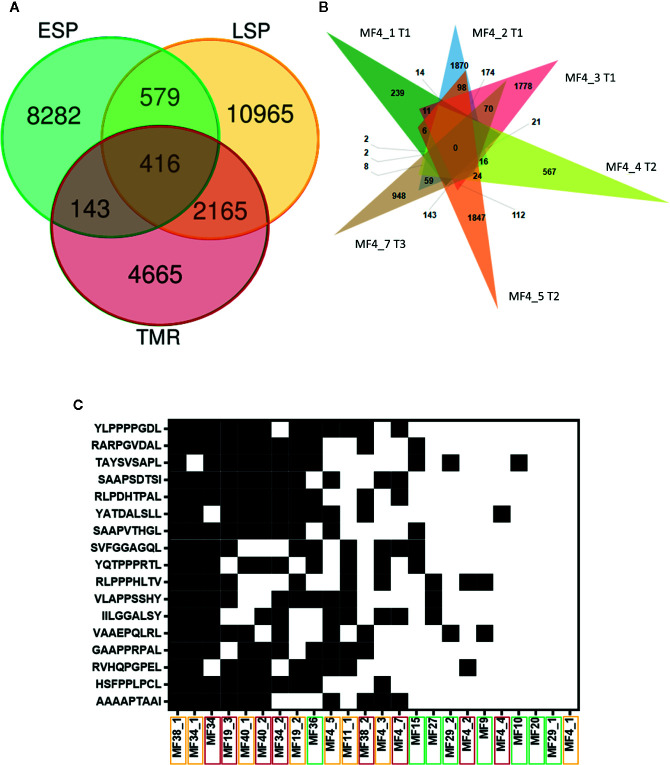
Intraindividual and interindividual overlap of neoantigens. **(A)** Each lesion type comprises eight samples, of which only unique peptides are included. The greatest overlap in filtered neoantigens is between plaques (LSP) and tumors (TMR). Early stage plaques (ESP) are also shown. **(B)** Venn diagram of filtered neoantigens from 6 samples obtained from one patient. Each sample name is accompanied by the time point the biopsy was obtained (initial biopsy at T1, T2 at 9 months after T1 and T3 10 months after T1). There is no overlap in peptides between all lesions, and the predominant exclusivity of peptides to their individual sites indicates the highly branched nature of the tumor. **(C)** Filtered neoantigens predicted in 10 or more samples out of the total 24 samples. Black indicates the presence of the peptide in the sample and white indicates the absence. Peptides are arranged from highest frequency (top) to lowest frequency (bottom). Sample names are arranged in order of those with the most overlapping neoantigens (left) to the least overlapping neoantigens (right). Sample names are enclosed in boxes with colors corresponding to the lesion type - ESP (green), LSP (yellow), and TMR (red). Overlapping neoantigens are mostly in the advanced stage disease samples (LSP and TMR) clustered on the left.

We examined the overlap in filtered neoantigens by lesion type (**Figure 6A**) and within the same patient sampled longitudinally (**Figure 6B**). The overlap between late stage plaques (LSP) and tumors (TMR) was greater than between early stage plaques (ESP) and either lesion type. This was expected, as we separated advanced disease into LSP and TMR for analysis in our study. We also examined neoantigens from one patient from whom multiple, longitudinal samples were obtained. Among six samples (three TMR, three LSP) obtained at three time points (0, 9, and 10 months respectively), we found no overlap in filtered neoantigens (**Figure 6B**). Most peptides were unique to each plaque or tumor site, further underscoring the predominantly subclonal structure of neoantigens in advanced disease. Finally, we examined the overlap of filtered neoantigens across samples (**Figure 6C**). No neoantigens were common to all samples, and the most common neoantigen was present in half of the 24 samples. Overlapping neoantigens were mostly present in late stage plaques and tumors. This is likely because advanced disease samples produced more neoantigens overall, increasing the likelihood of overlap.

Using the neoantigens we found, we searched IEDB for closely related peptides from humans or human pathogens (**Supplementary Table S4**). These known immune epitopes have been tested in experimental assays and are likely to elicit immunogenic responses in humans. We included epitopes tested in T-cell, B-cell and MHC ligand assays and did not require assays to be positive. Only 2 neoantigens were positive in T-cell assays.

## Discussion

We have previously demonstrated that as MF progresses from early to advanced stages, the tumor accumulates somatic mutations and evolves to produce multiple genetic subclones (20). The impact of this genetic diversity on tumor immunogenicity is two-fold. An increase in mutation load would result in higher neoantigen expression and increased opportunities for the neoplasm to be recognized by the immune system. Conversely, the increasing subclonal distribution of neoantigens would direct the immune system to discrete subpopulations of the most immunogenic tumor cells. This in turn would shield the less immunogenic subclones from the antitumor attack (42).

In this study, which to our knowledge is the first analysis of neoantigens in MF, we found that the neoantigen load mirrors the mutational load of MF and increases during disease progression. Our experimental approach using microdissected tumor tissue and deep exome sequencing allowed for identification of a markedly higher number of non-synonymous mutations (median 3,217) than previous MF studies (42-102) (26, 43, 44). The neoantigen load in our MF samples was also higher than other malignancies known to have a high neoantigen load such as malignant melanoma (median 121) and lung adenocarcinoma (median 335) (45). The differences are not only quantitative, as we were able to detect numerous frameshift mutations (median 2,604) which have hardly been captured in previous studies. Frameshift mutations are an essential source of neoantigens because they often produce highly immunogenic peptides due to global structural aberrations that render the peptide dissimilar from self (46, 47). Overall CTCL is known to have a high number of chromosomal aberrations and protein fusion is likely an additional source of neoantigens (48), that should be studied in the future. Thus, MF can be viewed as a neoplasm of high immunogenic potential expressing a significant number (median 328) of high strength neoantigenic peptides.

Analysis of the subclonal heterogeneity of the neoantigens by bioinformatic deconvolution of phylogenetic trees and by multisampling distinct lesions of MF revealed a complex neoantigenic landscape. Our analysis demonstrated that different cutaneous lesions of MF exhibit highly diverse repertoires of non-overlapping neoantigens. It has been previously demonstrated that multiple longitudinal CTCL biopsies from a single patient show molecular heterogeneity (49). Likewise, our analysis of six lesions from a single patient (**Figure 6B**) did not demonstrate a single shared antigenic peptide. Similarly, the overlap between neoantigens in plaques and tumors from the same patient was poor. Thus, a single patient with MF presenting with numerous skin lesions may be considered as having a collection of multiple, immunologically different neoplasms.

Not only did different lesions vary by their neoantigens but significant neoantigenic heterogeneity was also detected in different lymphoma subclones. Using a bioinformatic approach we were able to show that a large proportion of neoantigens map to the subclones (clades) and that this proportion increased during stage progression. Although it is tempting to speculate that this high proportion of subclonal neoantigens will render advanced stage MF resistant to immunotherapy (21), we have to acknowledge certain limitations of our computational approach. The phylogenetic trees were constructed by statistical modeling of point mutation distributions in the sample and were not verified by single-cell sequencing. Therefore we cannot with certainty equate a branch of the phylogenic tree with a clone of tumor cells.

Although there was a clear increase in the number of neoantigens between early stage plaques and lesions in the late stage disease, it has not escaped our attention that the clinically more advanced lesions of tumors did not have a higher number of antigens (some even had a lower neoantigen load) compared to late stage plaques. This could not have been explained by a lower degree of genetic heterogeneity because the tumors had a highly branched subclonal architecture. We hypothesize that the reduction in neoantigen expression might be a result of immune editing, whereby the cells bearing the most immunogenic neoantigens are negatively selected by the immune system (50). To gain further insight into the significance of the neoantigen landscape as a biomarker of response to immunotherapies we calculated the CSiN indexes which provide a simple measure of cancer immunogenicity. Similar to what was shown previously (40), the CSiN scores did not correlate with TMB and did not predict the risk of stage progression. We found however that a higher proportion of advanced lesions (LSP and TMR) have lower, unfavorable scores (CSiN<1) predictive of poor response to checkpoint inhibitor treatment. This may explain why a significant proportion of MF patients do not respond to immunotherapy (16). On the other hand, more CSiN scores >1 were found in the early MF lesions which makes those patients obvious candidates for target enrichment trials with immune checkpoint inhibitors.

Previous studies have reported that very few neoantigens are shared across patients in high mutation load malignancies (45) and as already mentioned, our cohort of MF patients did not share any neoantigenic peptides. However, several peptides were commonly found in some patients (**Figure 6C**) and these could represent potential therapeutic targets. We therefore searched for known homologous immune epitopes of the most frequently observed neoantigens (51). Although none of the homologous sequences were an exact match to our mutant peptides, there were numerous promising partial matches (90% sequence similarity) to immunogenic human sequences and the sequences of human pathogens such as *Mycobacterium tuberculosis* and protozoa (*Leishmania* and *Trypanosoma*) (**Supplementary Table S4**). This observation was particularly interesting because neoantigenic peptides homologous to human pathogens are known to be robust activators of the immune response (34, 52). However, the relevance of these peptides are unclear as these organisms are uncommon to Canada and Denmark, from where patients were recruited. Other notable homologous epitopes included those from proteins implicated in other cancers, such as the ENA family (involved in cell motility and adhesion) from breast cancer (53), and baculoviral IAP repeat-containing protein 6 (involved in anti-apoptosis through caspase inhibition) from brain cancer (54). Future studies should validate candidate neoantigen expression at the protein level and their ability to elicit T-cell activation.

In conclusion, we have shown a bewildering degree of neoantigen heterogeneity in MF. Among hundreds of detected strong neoantigens there is little overlap between different individuals, between lesions in the same individual and between different subclones within the same lesion. We hypothesize that neoantigen heterogeneity may be an important factor limiting efficacy of immunotherapy in MF, and probably in other highly mutated, genetically heterogeneous cancers.

## Data Availability Statement

The datasets presented in this study can be found in online repositories. The names of the repository/repositories and accession number(s) can be found below at: database of Genotypes and Phenotypes (dbGAP), https://www.ncbi.nlm.nih.gov/gap/, phs001877.v1.p1.

## Ethics Statements

The studies involving human participants were reviewed and approved by Health Research Ethics Board of Alberta. The patients/participants provided their written informed consent to participate in this study. No animal studies are presented in this manuscript. No potentially identifiable human images or data is presented in this study.

## Author Contributions

AS contributed to the study design, conducted bioinformatics, analyzed the data, and wrote the manuscript. DH conducted the bioinformatics analysis. ZX and DH performed the CSiN calculations. AI and SO performed the wet lab experiments. AS, PS, ZX, and GV prepared the figures. RG designed the experiments, supervised the data analysis, and edited the manuscript. All authors contributed to the article and approved the submitted version.

## Funding

This study was supported by grants from the following sources: Canadian Dermatology Foundation (CDF RES0035718), University Hospital Foundation (University of Alberta), Bispebjerg Hospital (Copenhagen, Denmark), Danish Cancer Society (Kræftens Bekæmpelse R124-A7592 Rp12350) and an unrestricted research grant to RG from the Department of Medicine, University of Alberta. AS was supported by scholarships from the Canadian Institutes of Health Research (CIHR), Alberta Innovates, and the University of Alberta.

## Conflict of Interest

The authors declare that the research was conducted in the absence of any commercial or financial relationships that could be construed as a potential conflict of interest.
