# Supplementary material for: The Neoantigen Landscape of Mycosis Fungoides
Source: Front Immunol. 2020 Nov 23;11:561234. doi: 10.3389/fimmu.2020.561234 (PMC7719792; doi:10.3389/fimmu.2020.561234)
Supplement: Supplementary file 1 [file DataSheet_1.docx]

Supplementary Material

**Table S1: Characteristics of patients and samples used in the study**

| **Patient ID (age, sex, race)** | **Sample ID** | **Lesion type** | **Location** | **Diagnosis & stage** |
| --- | --- | --- | --- | --- |
| MF 4 (69, M, Caucasian) | MF4_1P | Plaque (Late stage) | Forearm | Mycosis fungoides IIB |
|  | MF4_2T | Tumour | Flank |  |
|  | MF4_3P | Plaque (Late stage) | Shin/leg |  |
|  | MF4_4T | Tumour | Trunk |  |
|  | MF4_5P | Plaque (Late stage) | Leg |  |
|  | MF4_7T | Tumour | Neck |  |
| MF9 (42, F, Caucasian) | MF9P | Plaque (Early stage) | Trunk | Mycosis fungoides IA |
| MF10 (56, M, Caucasian) | MF10P | Plaque (Early stage) | Triceps region/arm | Mycosis fungoides IB |
| MF11 (56, M, Caucasian) | MF11_1P | Plaque (Late stage) | Leg | Mycosis fungoides IIB |
| MF15 (65, M, Caucasian) | MF15P | Plaque (Early stage) | Leg | Mycosis fungoides IB |
| MF19 (74, M, Caucasian) | MF19_2P | Plaque (Late stage) | Arm | Mycosis fungoides IIB |
|  | MF 19_3T | Tumour | Trunk |  |
| MF20 (70, M, Caucasian) | MF20 | Plaque (Early stage) | Trunk | Mycosis fungoides IB |
| MF27 (71, M, Caucasian) | MF27P | Plaque (Early stage) | Buttock | Mycosis fungoides IA |
| MF29 (87, F, Caucasian) | MF29_1P | Plaque (Early stage) | Neck | Mycosis fungoides IA |
|  | MF29_2P | Plaque (Early stage) | Foot |  |
| MF34 (65, M, Caucasian) | MF34T | Tumour | Abdomen | Mycosis fungoides IIB |
|  | MF34_1P | Plaque (Late stage) | Abdomen |  |
|  | MF34_2T | Tumour | Arm |  |
| MF36 (64, M, Caucasian) | MF36P | Plaque (Early stage) | Thigh | Mycosis fungoides IA |
| MF38 (76, M, Caucasian) | MF38_1P | Plaque (Late stage) | Abdomen | Mycosis fungoides IIB |
|  | MF38_2T | Tumour | Chest |  |
| MF40 (59, F, Caucasian) | MF40_1P | Plaque (Late stage) | Axilla | Mycosis fungoides IIB |
|  | MF40_2T | Tumour | Axilla |  |

**Table S2: Sequencing depth of individual samples**

| **Sample ID** | **Lesion type** | **Sequencing depth (x)** |
| --- | --- | --- |
| MF4_1P | Plaque (Late stage) | 140.5 |
| MF4_2T | Tumour | 192.0 |
| MF4_3P | Plaque (Late stage) | 183.8 |
| MF4_4T | Tumour | 190.0 |
| MF4_5P | Plaque (Late stage) | 111.2 |
| MF4_7T | Tumour | 155.8 |
| MF9P | Plaque (Early stage) | 146.9 |
| MF10P | Plaque (Early stage) | 122.1 |
| MF11_1P | Plaque (Late stage) | 180.7 |
| MF15P | Plaque (Early stage) | 199.5 |
| MF19_2P | Plaque (Late stage) | 186.3 |
| MF 19_3T | Tumour | 197.4 |
| MF20 | Plaque (Early stage) | 138.6 |
| MF27P | Plaque (Early stage) | 179.1 |
| MF29_1P | Plaque (Early stage) | 112.3 |
| MF29_2P | Plaque (Early stage) | 100.1 |
| MF34T | Tumour | 171.3 |
| MF34_1P | Plaque (Late stage) | 149.8 |
| MF34_2T | Tumour | 203.7 |
| MF36P | Plaque (Early stage) | 145.3 |
| MF38_1P | Plaque (Late stage) | 170.8 |
| MF38_2T | Tumour | 168.5 |
| MF40_1P | Plaque (Late stage) | 196.9 |
| MF40_2T | Tumour | 184.3 |

**Table S3: Characteristics of CTCL studies used in meta-analysis**

| **Study** | **Sample type** | **Sequencing method** | **Sequencing depth (x)** | **Number of samples** |
| --- | --- | --- | --- | --- |
| Choi et al. | Sézary Syndrome | Whole exome sequencing | Range 142.219-333.623 | 31g |
| McGirt et al. | Mycosis fungoides | Whole genome sequencing | Range 32.04-44.24 | 5 |

**Table S4: Homologous immune epitopes of filtered neoantigens.** Only epitopes from humans or human pathogens were included. Included are epitopes tested in T-cell, B-cell and MHC ligand assays. There was no requirement that assays be positive. Peptides highlighted in green were positive in T-cell assays.

**
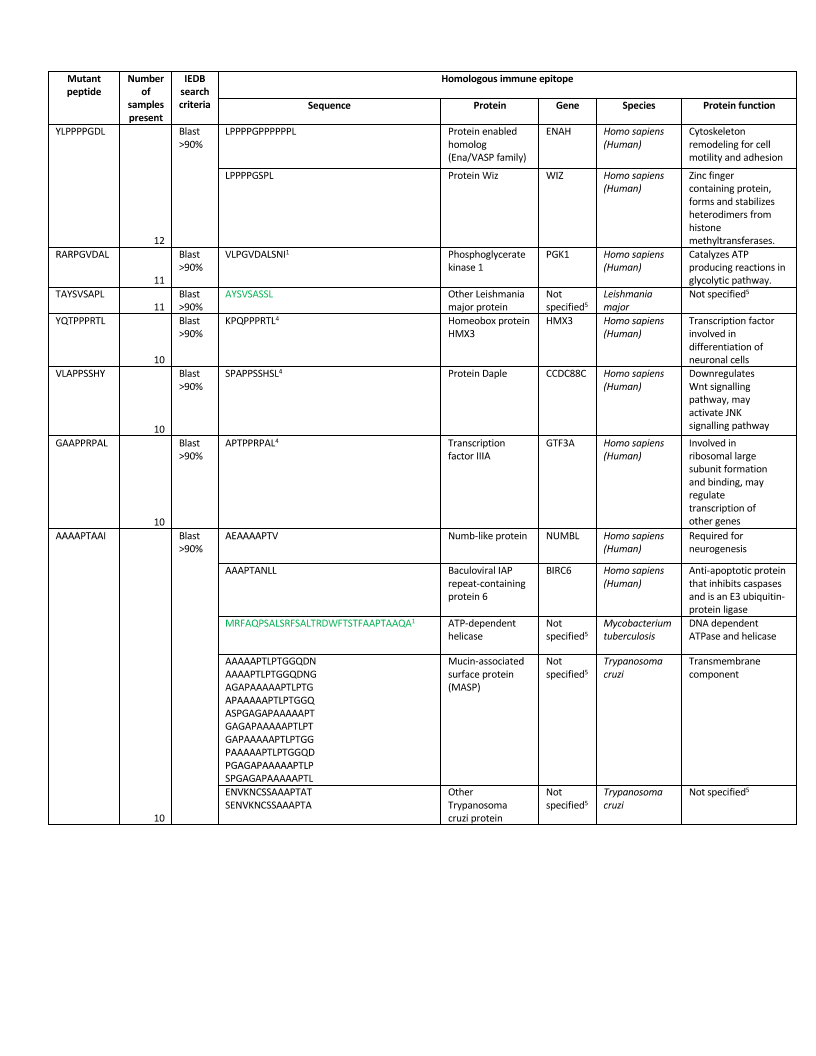
**

The following peptides did not yield any relevant search results: SAAPSDTSI, RLPDHTPAL^2^, YATDALSLL, SAAPVTHGL^3^, SVFGGAGQL, RLPPPHLTV^3^, IILGGALSY^2^, VAAEPQLRL, RVHQPGPEL and HSFPPLPCL^3^.

^1^ Multiple homologous peptide sequences were found from the same protein or organism. The sequence tested in the most assays is listed here.

^2^ The homologous sequence found had incomplete information and was excluded.

^3^ The homologous sequence was from an organism that is not a typical human pathogen.

^4^ Another homologous protein was excluded due to being from an organism that is not a typical human pathogen.

^5^ “Not specified” indicates not enough distinguishing information was provided in the IEDB database to search peptide features.

**Figure S1: Comparisons of neoantigens from our datasets with those of Choi at al.** [(27)](https://paperpile.com/c/Qeuqd5/npwp) **and McGirt et al.** [(26)](https://paperpile.com/c/Qeuqd5/vk2e) This beeswarm plot shows putative neoantigens prior to filtering. Due to the extensive size of the dataset, a random 1% of all data points were plotted to demonstrate the overall distribution and density of the data. The vertical axis shows mutant peptide binding strength as a percentile rank, with lower values representing increasingly strong binding peptides to HLA types. 0.5% rank (dashed line) represents the commonly used cutoff below which peptides are considered strong enough binders to be neoantigens. The horizontal axis shows the three studies with the number of total putative neoantigens in each dataset specified in brackets. The width of each plot represents the quantity of neoantigens at each binding strength. Overall, our dataset had a many fold greater number of putative neoantigens compared to the Choi and McGirt datasets. The slight difference in median binding strength is likely due to the vastly greater size of our dataset (57% rank) compared to the Choi dataset (52% rank) and the McGirt dataset (52% rank). For the McGirt and Choi datasets, there was no RNA data or separation by lesion stages. Consequently, the median filtered neoantigen load was 40-46 per sample (supplementary **Figure S2&S3**)


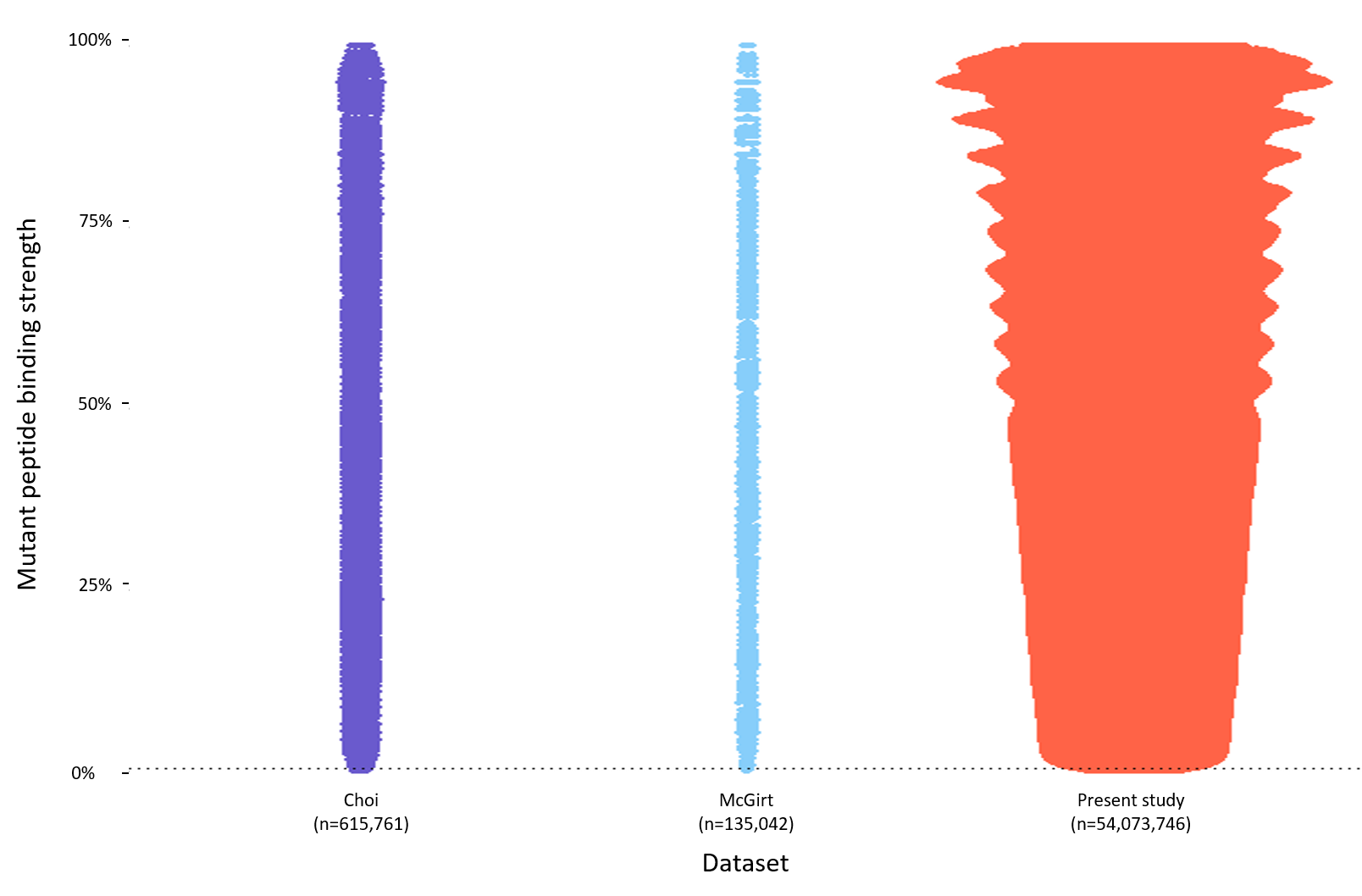


**Figure S2: Characteristics of the dataset from Choi et al.** [(27)](https://paperpile.com/c/Qeuqd5/npwp)**. A:Tumour mutation burden.** Samples are arranged in descending order of TMB. Missense mutations comprise 96% of the non-synonymous mutations. **B: Filtered neoantigen load.** All filters were applied with the exception of the RNA filter as expression data was not available. The median number of filtered neoantigens per sample was 40.


**Figure S3: Characteristics of the dataset from McGirt et al.** [(26)](https://paperpile.com/c/Qeuqd5/vk2e)**. A:Tumour mutation burden.** Samples are arranged in descending order of TMB. Missense mutations comprise 98% of the non-synonymous mutations. **B: Filtered neoantigen load.** All filters were applied with the exception of the RNA filter as expression data was not available. The median number of filtered neoantigens per sample was 46.
